# Giant Robber Crabs Monitored from Space: GPS-Based Telemetric Studies on Christmas Island (Indian Ocean)

**DOI:** 10.1371/journal.pone.0049809

**Published:** 2012-11-14

**Authors:** Jakob Krieger, Ronald Grandy, Michelle M. Drew, Susanne Erland, Marcus C. Stensmyr, Steffen Harzsch, Bill S. Hansson

**Affiliations:** 1 Ernst-Moritz-Arndt-University of Greifswald, Zoological Institute and Museum, Cytology and Evolutionary Biology, Greifswald, Germany; 2 Max Planck Institute for Chemical Ecology, Department of Evolutionary Neuroethology, Jena, Germany; University of Arizona, United States of America

## Abstract

We investigated the navigational capabilities of the world's largest land-living arthropod, the giant robber crab *Birgus latro* (Anomura, Coenobitidae); this crab reaches 4 kg in weight and can reach an age of up to 60 years. Populations are distributed over small Indo-Pacific islands of the tropics, including Christmas Island (Indian Ocean). Although this species has served as a crustacean model to explore anatomical, physiological, and ecological aspects of terrestrial adaptations, few behavioral analyses of it exist. We used a GPS-based telemetric system to analyze movements of freely roaming robber crabs, the first large-scale study of any arthropod using GPS technology to monitor behavior. Although female robber crabs are known to migrate to the coast for breeding, no such observations have been recorded for male animals. In total, we equipped 55 male robber crabs with GPS tags, successfully recording more than 1,500 crab days of activity, and followed some individual animals for as long as three months. Besides site fidelity with short-distance excursions, our data reveal long-distance movements (several kilometers) between the coast and the inland rainforest. These movements are likely related to mating, saltwater drinking and foraging. The tracking patterns indicate that crabs form route memories. Furthermore, translocation experiments show that robber crabs are capable of homing over large distances. We discuss if the search behavior induced in these experiments suggests path integration as another important navigation strategy.

## Introduction

Monitoring freely roaming animals in their natural habitat is not an easy task. Doing so for extended periods of time is even trickier. Animals are typically shy, and the very act of observation may also affect their behavior. The invention of space-based satellite navigation systems, such as the NAVSTAR Global Positioning System, or GPS for short, and the subsequent miniaturization of receiver and transmitter units, have revolutionized the field of animal tracking. This technology, which allows animals' behavior to be unobtrusively monitored, has provided valuable information on long-distance migration [1]–[3] and key insights into the daily activity patterns of many animals as they move around in their habitat.

Although it has revolutionized the field of vertebrate behavior, GPS technology has yet to reach the most abundant group of animals on earth: the arthropods, or other invertebrates. The large size of most GPS technology trackers has limited its use in understanding the behavior of small arthropods. Methods employed to follow individual arthropods through time and space apart from direct observation using grids [4]–[6], have included mark-recapture approaches [7], [8] and the use of radar [9]. However, none of these methods, or any other methods tried, affords the combination of constant long-term tracking and high-resolution monitoring that space-based satellite navigation systems offer.

**Figure 1:**
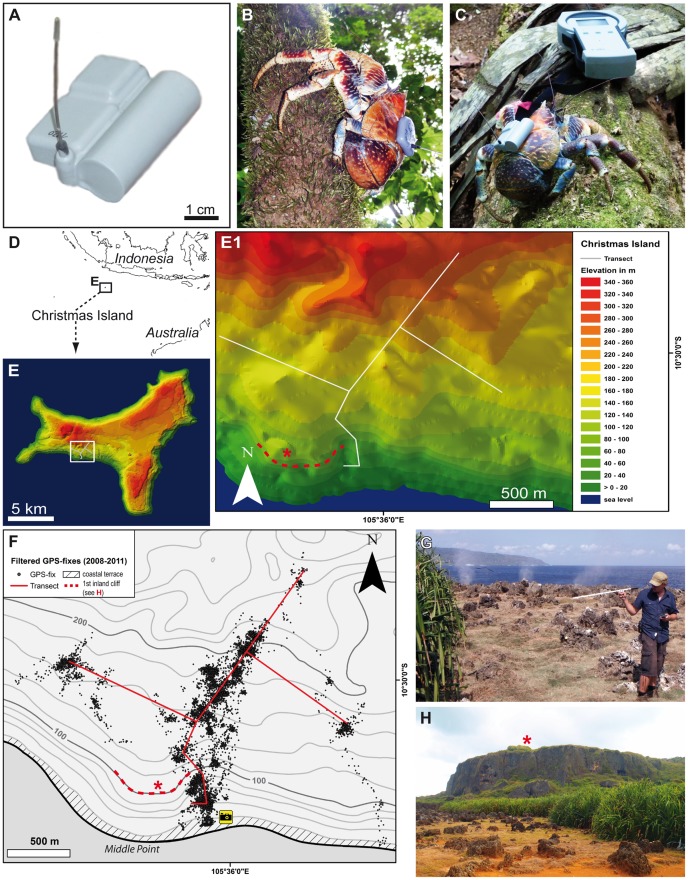
Animals and tags. **A:** Photograph of the e-obs GPS-tag (2^nd^ tag version with single battery used in 2010 and 2011). **B** Tagged *Birgus latro* escaping into a tree (1^st^ tag version with twin batteries used in 2008). **C** Tagged *B. latro* (2^nd^ tag version) inspecting the BaseStation for wireless data download (image kindly provided by Meike Kilian). **D** Location of Christmas Island, Indian Ocean. **E,** **E1** Geo-referenced 3D model of Christmas Island showing the study area at Aldrich Hill and the transect. **F** Topographic map displaying the filtered data set of 9,272 GPS fixes recorded in all three expeditions. The camera symbol shows the position from which G and H were taken. **G, H** Photographs of the coastal terrace. G shows the first author tracking radio signals near the blowholes which can be identified by the three spray plumes in the background (image kindly provided by Meike Kilian). H is taken facing north-west and the photo shows the rocky coastal terrace, the dense belt of *Pandanus* and in the background the vertical first inland cliff. For positions of asterisk, see F.

Here for the first time GPS tagging is used in large-scale experiments to track an invertebrate in its natural environment, namely the giant robber crab *Birgus latro* (Linnaeus, 1767) (Malacostraca, Anomura). These animals which are able to open and consume coconuts [10] – are the world's largest extant land-living arthropods (Fig. 1 B, C). Robber crabs can weigh up to 4 kg, have a leg span of almost 1 m [11] and reach an age of up to 60 years [10]. ‘Formerly common on tropical islands of the Pacific and Indian Ocean, populations of robber crabs in most island locations are now declining. Robber crabs are essentially oversized hermit crabs (Anomura), and are only dependent on water for the pelagic larval stages [12]–[14]; in fact, adult robber crabs have lungs and drown if immersed in water for more than 24 hours [15]. Despite their terrestrial nature and large size, we know little about their biology. Of note is the lack of knowledge of their migratory behavior and its underlying orientation mechanisms. Such long-lived animals, are likely to acquire a good knowledge of their environment and the navigational skills needed to move between places of interest, such as shelters, access to conspecifics, food and water, but so far, only basic aspects of short-distance movements have been analyzed [16].

Although the behavior of the robber crabs is poorly understood, certain behavioral aspects of other crustaceans have been studied in quite some detail. The marine spiny lobsters of the genus *Panulirus* are famous for their autumnal and storm-associated synchronous long-distance migrations, which can span over several hundred kilometers (e.g. [17]–[20], [7]). Clawed lobsters (Homarida) are known to seasonally migrate (e.g. [7], [8]). During non-migratory periods, lobsters also show a strong homing instinct to their dens. There are, however, severe difficulties associated with investigating aquatic crustacean behavior, chiefly, working underwater. Therefore, the numerous crustacean lineages that have successfully invaded land offer more feasible research possibilities (e.g. [14], [21]). Semi-terrestrial beach hoppers (Talitridae, Amphipoda), mangrove-dwelling grapsid crabs (*Sesarma leptosoma*), terrestrial hermit crabs (Coenobitidae), fiddler crabs (*Uca* ssp.) and Christmas Island red crabs (*Gecarcoidea natalis*) have all been the subjects of investigations within their habitats (reviews [17], [18], [22]), and in some cases navigational mechanisms have been proposed (reviews [23]–[25]). Although these studies have provided important insights into crustacean behavior specifically, and arthropod behavior in general, studies based on non-automated observation suffer from a poor recovery rate (mark-recapture techniques), or low levels of temporal and/or spatial resolution because direct observation or manual telemetry over longer periods (weeks or months) is arduous. Automated systems can overcome some of these shortcomings. Recently, small-scale movements of clawed lobsters (Homarida) in their habitats were monitored by automated ultrasonic fixed array telemetry, which makes use of arrays of buoys that triangulate the position of tagged lobsters under semi-natural conditions in an enclosure (mesocosm [26]; see also [27], [28] for details of this method). However, long-term data on what crustaceans actually do, how they move around and what places they know are scarce.

The terrestrial life-style, size, and physical strength of the robber crabs make these long-lived animals ideal subjects for exploration *via* GPS tracking. We examined the behavior of freely moving crabs on Christmas Island (Indian Ocean), which holds one of the largest and mainly undisturbed remaining populations of this species (Fig. 1D and E). In the present paper, we provide details of the locomotory activity, routes of movement, and orientation strategies used by this arthropod, obtained over several months of monitoring. We demonstrate that male robber crabs display long periods (weeks) of exclusive site fidelity to their home but also perform directed, long-distance migrations over several kilometers. Translocation experiments further show that robber crabs are capable of homing over large distances. We discuss possible reasons for this behavior and the navigational strategies used.

## Materials and Methods

### Study site

Christmas Island is a relatively large oceanic island located approximately 360 km south of Java, Indonesia, in the Indian Ocean (Fig. 1D). The island originated from an ancient reef base that has been uplifted over time [29]. The landmass is characterized by a series of stepped plateaus (the central, middle, and coastal plateau) which are separated by very rugged cliffs.

Crabs were tagged and monitored in the vicinity of Aldrich Hill (10°30′06″S, 105°36′04″E; Fig. 1 D), within the Christmas Island National Park. The study site, which extended from the mid-plateau to the coastal plateau near Middle Point (ca. 260 m above sea level), intercepted a number of different habitats, ranging from open rainforest on deep soil to thick stands of *Pandanus sp*. growing on shallow soils over limestone and various small cave systems. The study area traversed a number of gentle slopes on the mid-plateau before descending a steep slope to the coastal plateau and ocean cliffs (ca. 10 m above sea level).

Tagging and monitoring of the crabs followed several four-wheel drive vehicle tracks currently maintained for Parks Australia access. Access to the coastal plateau from the mid-plateau was conducted on foot, as no off-road tracks were maintained to Middle Point. The northern end of the study area was accessed *via* the north-west baseline and was approximately 2.5 km in length (north-south). Crabs were also monitored in easterly and westerly directions following off-road tracks (Fig. 1), with a total distance of 2 km. Additional incursions into the forest were often conducted to attempt to relocate crabs based on last GPS fixes. The estimated area of the study site was approximately 3 km^2^ assuming an optimal receiving range for data acquisition of 300 m (as field-tested on site).

### Tags and animals

The GPS-RF-tags (*N* = 61; Fig. 1A) were manufactured by e-obs digital telemetry in Grünwald, Germany (http://www.e-obs.de) and consisted of a power supply (lithium polymer battery cell with 4.5 V); a flash memory SD-card; a GPS module (LEA 4S by u-blox™); a radio transmitter (“pinger”), which transmitted short and high-pitched signals at brief intervals on idiosyncratic frequencies; an on-board real time clock; an antenna; and an interface for an RF link to the BaseStation, a mobile interface between user and GPS-RF-tag (BaseStation b5, e-obs). All components were embedded into a hard, waterproof plastic housing; tags measured 6 cm in length, 1.5 cm in height and 5 cm in width and weighed ca. 57 g.

All monitoring was conducted with permission from Christmas Island National Parks, Parks Australia North (permit numbers: AU-COM2008043, AU-COM2010090, and AU-COM2011106). All individuals captured were photographed, measured, weighed, and tagged. The GPS positions of the tagging sites and other important landmarks like the transect dimensions were documented with a handheld GPS receiver (Garmin GPSMAP®62; http://www.garmin.com).

**Figure 2:**
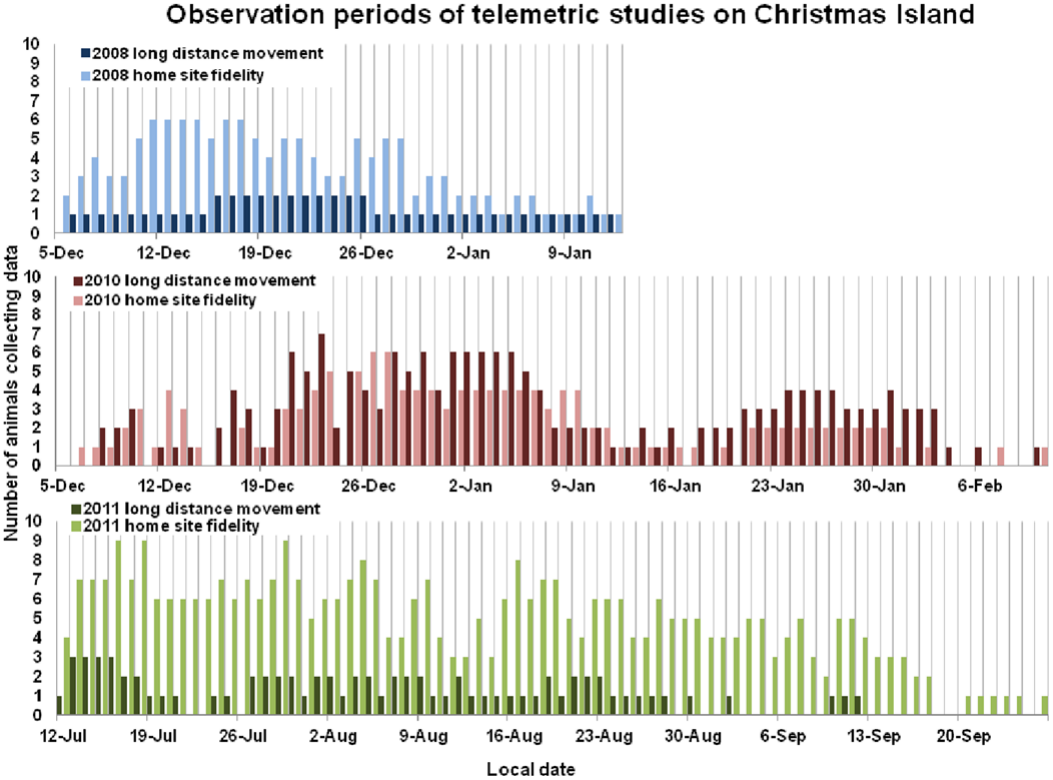
Observation periods of telemetric studies on Christmas Island. Colored bars indicate the number of animals/transmitters receiving at least one GPS fix per day. Stationary (brighter colored bars) animals are opposed to animals showing large-distance migrations (darker colored bars) daywise (for details see text).

Tags were attached to the posterior part of the dorsal carapace of *B. latro* with industrial two-component epoxy resin (Araldite® 2012 EP) after gridding and degreasing the surface with sandpaper and acetone (Fig. 1B, C). Only male crabs greater than 500 g in weight were tagged (*N* = 42; range 600 to 2,940 g). The tag-to-body weight ratio was 4.0±1.9% (range 1.9 to 9.5%), which is within the generally accepted range of <3 to 5% for telemetric studies [30]. *Birgus latro* has remarkable body strength with a reported lifting force of up to 28 kg [31], enabling it to effectively climb trees (Fig. 1B). Therefore, and because Kenward [30] referred mainly to the tagging procedures of vertebrates, we assume that the proposed threshold is not applicable for land-living hermit crabs and can be easily increased up to 10 to 15% of body weight. Our observations during the tagging procedure support the claim that the weight of the tags does not impair the mobility of the animals. The tags were programmed to collect data at 30, 60, or 120 min intervals with an acquisition time of 180 s and 210 s in 2008 and 2010/11 respectively. After the first study in 2008, the tag settings and tagging procedure, and the routine of data collection, were continually optimized, resulting in an increase of maximum battery life-time from 38 days in 2008 up to 77 days in 2011 and an increase of data retrieval ratios (number of tags with data download/total tag number per year) from 71% in 2008 up to 95% in 2011 (for more details, see Table 1 and Fig. 2). The rate of successful GPS fixes per programmed GPS fixes was 33.34% in total.

**Table 1:** Data from three years of GPS telemetric experiments on Christmas Island.

Year	2008	2010	2011	total
programmed GPS fixes	7,729	17,813	24,370	49,912
received GPS fixes	4,360	4,726	6,207	15,293
receiving ratio in %	56.41%	26.53%	25.47%	30.64%
filtered GPS fixes	2351	2645	4276	9272
monitored crab days	164	547	962	1673
animals tagged	14	21	20	55
average animal weight in g (±SD)	n. a.	1,606±692	1,765±502	1,668±602*
average animal length in mm (±SD)	n. a.	58.7±9.4	60±7.6	59.3±8.5*
average animal width in mm (±SD)	142.5±24.8	144.8±23.5	134±15.3	140.3±22.9
translocated animals	0	4	8	12
average horizontal error estimate in m (±SD)	20.9±13.4	20.9±13.1	18.4±12.5	20±13.4

* *N* = 42 (only 2010 and 2011).

### BaseStation and radio receiver

The BaseStation featured a flash memory, a power supply, a display, a USB interface and an RF-interface for a wireless connection to the tag *via* a high-sensitivity antenna (developed and custom-made by Henning Marter FUNKBAU, Rudolstadt, Germany; www.funkbau.de). A conventional YAESU VR500 radio receiver was used to receive the “pinger” signal for each tag ranging from 868 to 867 MHz in 25 kHz steps with a directional Yagi-Uda antenna. For data acquisition, ca. every third day the data collected by the tags were downloaded along the transect until the tag batteries were discharged (maximum of 77 days).

### Displacement experiment

In addition to tagging undisturbed animals as described above, we conducted 12 displacement experiments during 2010 (*N* = 4) and 2011 (*N* = 8) to analyze possible homing behavior. In 2010, we translocated four male robber crabs in opaque jute bags approximately 1 km from north (10°29′59.77″S, 105°35′58.53″E; elevation 180 m) to south (10°30′31.44″S, 105°35′54.83″E; elevation 38 m). We repeated the experiment in 2011 with two animals taken from north (10°30′11.18″S, 105°35′50.57″E; elevation 150 m) approx. 750 m southward (10°30′31.44″S, 105°35′54.83″E; elevation 38 m) and translocated another two crabs in the opposite direction. One animal (No. 1717) from this group was displaced twice in the same manner after first returning successfully to the capture site. In addition to these translocations towards and away from the shore covering ca. 120 m of altitude, four animals were displaced roughly parallel to the coast within the same range of altitude as their capture location. Two animals were translocated from the transect (10°29′57.34″S, 105°36′10.97″E; elevation 189 m) one km ca. eastwards (10°30′11.10″S, 105°36′29.92″E; elevation 164 m) and two from the transect (10°30′11.18″S, 105°35′50.57″E; elevation 150 m) 750 m ca. westwards (10°29′55.39″S, 105°35′18.43″E; elevation 164 m).

### Analysis of spatial and telemetric data

All recorded data of GPS-RF tags (e.g. GPS date, GPS daytime, horizontal error estimate and heading direction) were transferred into a Microsoft® Excel table before being imported into ArcGIS® (ESRI) as a geo-database. To minimize GPS error, recorded GPS bursts of a maximum of four positions per GPS fix were averaged. For a precise measurement of movement, a 3D model of Christmas Island was created and geo-referenced with regard to the large variations in height above sea level (Fig. 1E). The model is based on a topographic map of Christmas Island (Natmap; edition 1; 1∶30,000; GEOCAT 70145). The tagging data were analyzed with ArcGIS® as well as the open source tools Geospatial Modelling Environment (http://spatialecology.com/gme) and Home Range Tools (HRT) [32]. Individual home ranges of *B. latro* were analyzed by using the quadratic Kernel function [33] with the least squares cross validation (LSCV) of the mean integrated square error [34].

### GPS accuracy and data filtering

**Figure 3:**
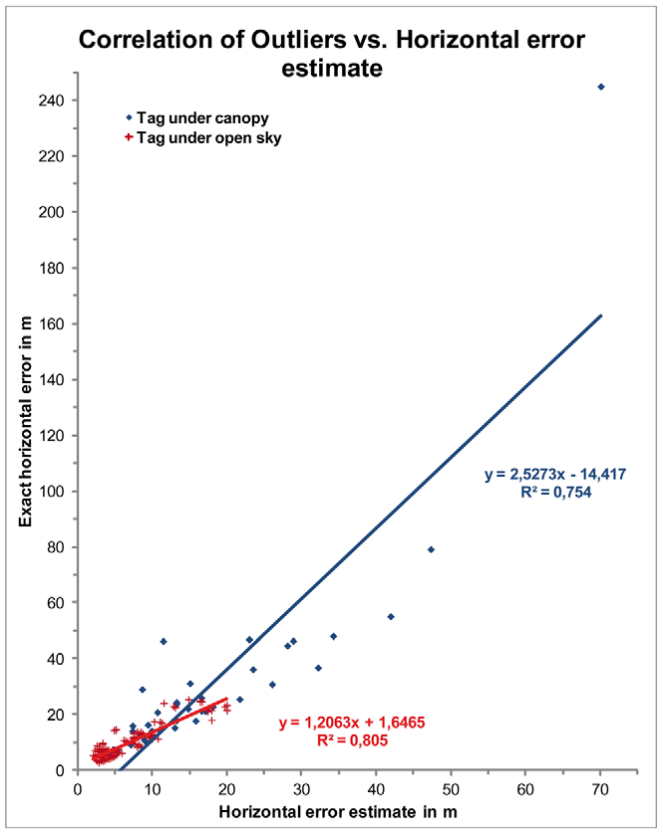
Linear correlation of horizontal error estimate and exact horizontal error. Horizontal deviations in m from stationary tags of known positions under open sky conditions (red crosses) and within the rain forest (blue diamonds) of Christmas Island.

The GPS-module calculates a horizontal error estimate by an algorithm based on the number and distance of used satellites. The GPS positioning accuracy was tested by placing one stationary tag under forest canopy to record 84 fixes over 44 h. The average horizontal error was 20.4±28.6 m (SD; range 2.3 to 244.74 m), with confidence intervals (CI) of CI50% = 19.31 m, CI95% = 56.06 m, and CI99% = 73.65 m. For comparison and for calibration of the GPS modules, the tag accuracy was also tested by placing 21 tags on a glade (open sky) near the upper end of the transect to record 669 fixes over 4 h. Here, the average horizontal error was 5.26±4.93 m (SD; range 0.02 to 27 m), CI50% = 3.32 m, CI95% = 9.65 m, and CI99% = 12.68 m. When the real horizontal error from a known position exceeds the horizontal error estimate, the values will be positive linearly correlated by a regression coefficient of determination of R^2^ = 0.8 (Fig. 3). The recorded accuracy of GPS fixes within the forest corresponds well with values from recent telemetric studies using GPS-tags in the rain forest of Central Panama [35]. Our entire data set was filtered by removing positions with horizontal error estimates exceeding 20.4 m, which is the average error in the forest, resulting in a total of 9,272 GPSfixes (61% of raw data) for the final analysis.

## Results

**Figure 4:**
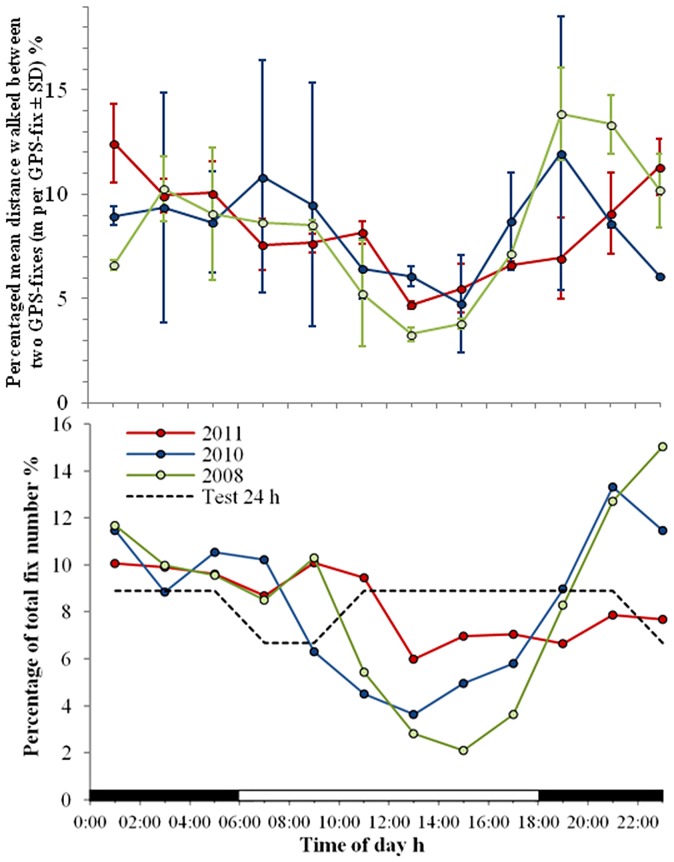
Circadian rhythm of locomotory behavior. Upper panel: Relative activity = percentage of mean distances between GPS-fixes per total number of GPS-fixes ± SD [percentage of m/fix]. Horizontal bars indicate times of daylight (white) and night (black). **Lower panel:** Daily fluctuations of GPS fixes of all attached tags of the 2008, 2010, and 2011 missions (colored solid lines) and a stationary test tag (black dashed line). Data suggest that many animals hide in refuges during daytime thus blocking GPS reception. During the 2008 and 2010 missions (wet season) migratory activity of the animals begins at dusk to reach a maximum between 20:00 and 22:00.

**Figure 5:**
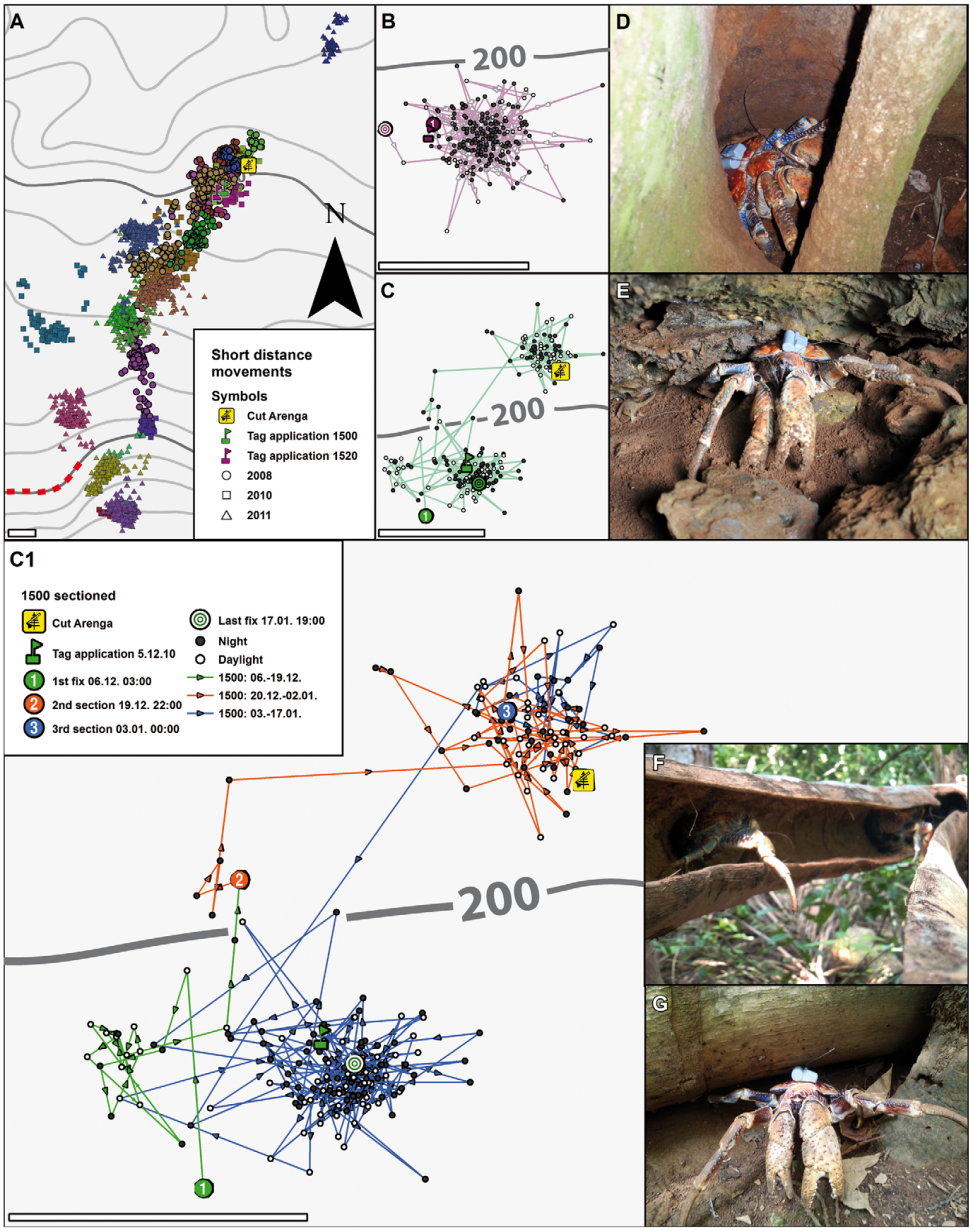
Site fidelity. **A:** Overview of all animals showing site fidelity and short-distance movements only from all three years of observations. **B:** Higher magnification of animal No. 1520 that remained within one home range during the plotted period (19 December 2010 to 09 January 2011); open symbols: daytime fixes, solid symbols: nighttime fixes. **C and C1:** higher magnifications of animal 1500 that occupied three home ranges between 5 December 2010 and 17 January 2011. In C1 this period is sectioned in three episodes. **D:** *B. latro* uses hollow trees as daytime refuges. **E:** A tagged animal hiding in a rock crevice during the day. **F, G:** *B. latro* are strongly attracted to freshly fallen Lister's palms. Cut Arenga: site of a freshly fallen *Arenga listeri* palm. Scale bars in A, B, C, C1: 100 m.

Our analysis is based on a filtered data set of 9,272 GPS positions (Fig. 1F) from 55 male animals (Table 1), some observed as many as 77 days, covering a total of 1,673 crab days. The data from stationary test tags showed slight, daily fluctuations of the GPS reception with fewer fixes received in the morning hours (Fig. 4). The number of GPS fixes obtained from the attached tags during the rainy seasons in 2008 and 2010 was constant during the night but decreased dramatically during morning hours. It was lowest around noon, rising again at dusk, and reaching a peak between 20:00 and 22:00 (Fig. 4). Based on field observations, we conclude that the animals are mostly night active. Because they hide in refuges such as rock crevices and shelters between tree roots during the daytime (Fig. 5), a sky view of the tags – and hence GPS reception – is blocked. During the dry season in 2011, the rise in activity at dusk was much less pronounced than in the wet seasons 2008 and 2010, suggesting that air humidity is a major factor limiting the animals' activity [10].

**Figure 6:**
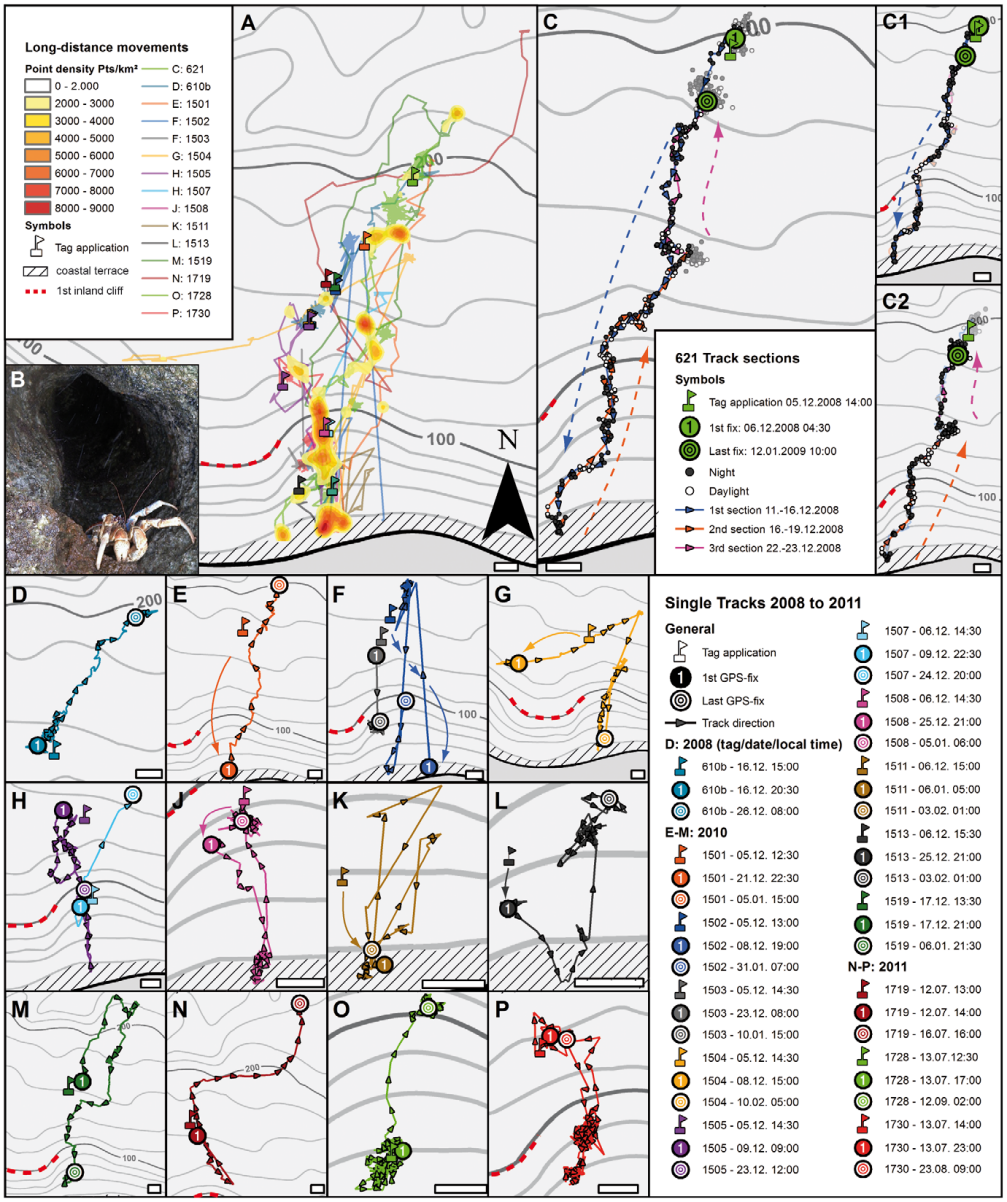
Long-distance Y-axis migrations. **A:** Overview of all animals showing long-distance movements within a migratory corridor that extends from the coastal terrace ca. 2 km inland (data pooled from all three missions). Color ranges define point density estimates in points per km^2^ using only data of directed movements (points of stationary phases or undirected movements were removed) **B:** One of several animals exposed to ocean spray as observed in the upper openings of the blowholes at the coastal terrace (compare Fig. 1J). **C:** example of migratory animal No. 621, monitored from 5 December 2008 to 12 January 2009, and displaying typical Y-axis migration (with the X-axis being the shoreline) during this period. C1 and C2 section this migration into the outbound and inbound episodes. Note that in- and outbound paths are identical (permitting for the GPS error of max. 20 m); open symbols: daytime fixes, solid symbols: nighttime fixes. **D–P:** higher magnifications of all animals showing long-distance movements. For every animal, position and date (see boxed legend) of the tag application is plotted as well as dates and positions of the first and last obtained GPS fixes. Scale bars: 100 m.

Our data show that *B. latro* males exhibit periods of considerable site fidelity interrupted by periods of directed migration. The two main behavioral patterns observed are:

1. Long periods (weeks) of exclusive home site fidelity, with short-distance excursions to one or several home areas with a radius of around 25 m (*N* = 28; Fig. 5).
2. Long-distance movements (*N* = 15; between 0.7 and 4.2 km) within a home range with interim stationary phases of home site fidelity (Fig. 6).

Here, the term “home site” is used for a roughly circular area around a temporary residence or refuge from which the animals undertake short-distance excursions, returning every day to the refuge. A “home range” is generally defined as the “area traversed by the individual in its normal activities of food gathering, mating and caring for young” [36] or simply, “an area repeatedly traversed by an animal” [30], and we use the term “home range” in this broad meaning.

### Site fidelity and short-distance movements

Animals exclusively expressing home site fidelity during the tracking period (Figs. 2 and 4; max. 77 days) undertook short-distance excursions mostly at night (Fig. 5B, C). This behavioral pattern was observed both inland at the northern reaches of our study site and on the coastal plateau. The average size (*N* = 27) of the areas occupied during these behavioral phases over all three years were ca. 0.2 ha (Kernel density KD50±0.2 ha; range: 0.03 to 0.85 ha); 1.0 ha (KD90±0.7 ha; range: 0.09 to 3 ha); and 1.2 ha (KD95±0.8 ha; range: 0.11 to 3.7 ha) based on the quadratic Kernel function [33] using the least square cross validation (LSCV) of the mean integrated square error for smoothing according to Worton [34] (for further details see [30]). Animals were observed to stay at one home site (Fig. 5B) or to change between several (up to three) (Fig. 5C). We found that activities were centered on refuges such as rock crevices, tree roots and holes in dead wood (Fig. 5D and E). Other important attracting factors were fruiting trees and cut wood of the endemic Lister's palm *Arenga listeri* (Beccari, 1891) around which crabs aggregated in large groups (Fig. 5F and G). The Lister's palm is known to be a strong attractant to *B. latro* [10], and during all expeditions we encountered feeding congregations surrounding palms. For example, shortly after tagging crab No. 1500 (Fig. 5C1), it was noted to move toward a recently fallen *A. listeri,* where it remained for ca. 2 weeks (orange episode) before returning to the tagging location (blue episode).

### Long-distance movements

Male robber crabs were observed performing long-distance movements within their home range, averaging 1.8±1.2 km (SD, *N* = 15; range: 0.7 to 4.2 km; excluding intermediate short-distance movements) between the inland rainforest and the coastal plateau during the wet season in 2008 and 2010. In contrast, only three long-distance movements were observed in the dry season (2011; Fig. 6N, O, P). Long-distance movements followed a strict coast-inland pattern (north-south in our case) and hence can be considered Y-axis movements in the sense of Herrnkind [18] where the X-axis corresponds to the coastline and the Y-axis to the direction of sea to land (Fig. 6A). We observed animals moving from south to north (Fig. 6D, E, H blue, N, O), from north to south (Fig. 5F, H violet), and back and forth (Fig. 6C, F blue, J, K, M), even multiple times (Fig. 6F blue, G, K), during the observation period of 67 days. These primarily nocturnal migrations occurred within a confined corridor of ca. 500 m width and covering ca. 200 m of altitude. Individual crabs were recorded moving up to 150 m/hour. The Y-axis migrations were frequently interrupted by almost stationary phases; for example, animal No. 1502 (Fig. 6F blue) spent ca. 39 days in the northern ranges of the transect between its two trips to the coastal plateau. The tracks indicate that within a migratory corridor between inland rainforest and coastal plateau, animals followed individual routes (Fig. 6A–P). Some animals were observed to use identical routes for seaward and landward migration (permitting for the GPS margin of error of ca. 20 m error; for further information, see Fig. 3). Animal No. 621, for example, having spent six days around the inland tagging site, walked from inland to the coastal terrace at Middle Point within five days (Fig. 6C1, blue arrow marks the direction of downhill track). After a day at the coast, it reversed its path to walk back to ca. 160 m of altitude where it remained for three days (Fig. 6C2, orange arrow). The animal then continued inland on the outbound path (Fig. 6C2, pink arrow) to a position some 200 m away from the original tagging site (Fig. 6C).

Close to the coast, the migratory corridor channeled across a steep but (for humans) walkable slope (altitude difference of ca. 80 m), which to the west was bordered by a vertical cliff (Fig. 1H) and to the east by a steep field of large rocks. Most of the seaward tracks led to a 50 to 100 m broad belt of screw-pines (*Pandanus* sp.) located on the coastal plateau (Fig. 6A). The coastal terrace of Middle Point is characterized by a steep cliff rising 5–15 m from the ocean. Extending inland from the ocean there is a 20–30 m wide band of extremely rugged limestone pinnacles, devoid of vegetation and cut by channels and fissures, many of which connect to the ocean, creating ‘blowholes’ during high seas. During the wet season in 2010, we observed many *B. latro*, including two tagged individuals, clinging to the upper reaches of the fissures that were frequently exposed to ocean spray (Fig. 6B).

### Displacement experiments

**Figure 7:**
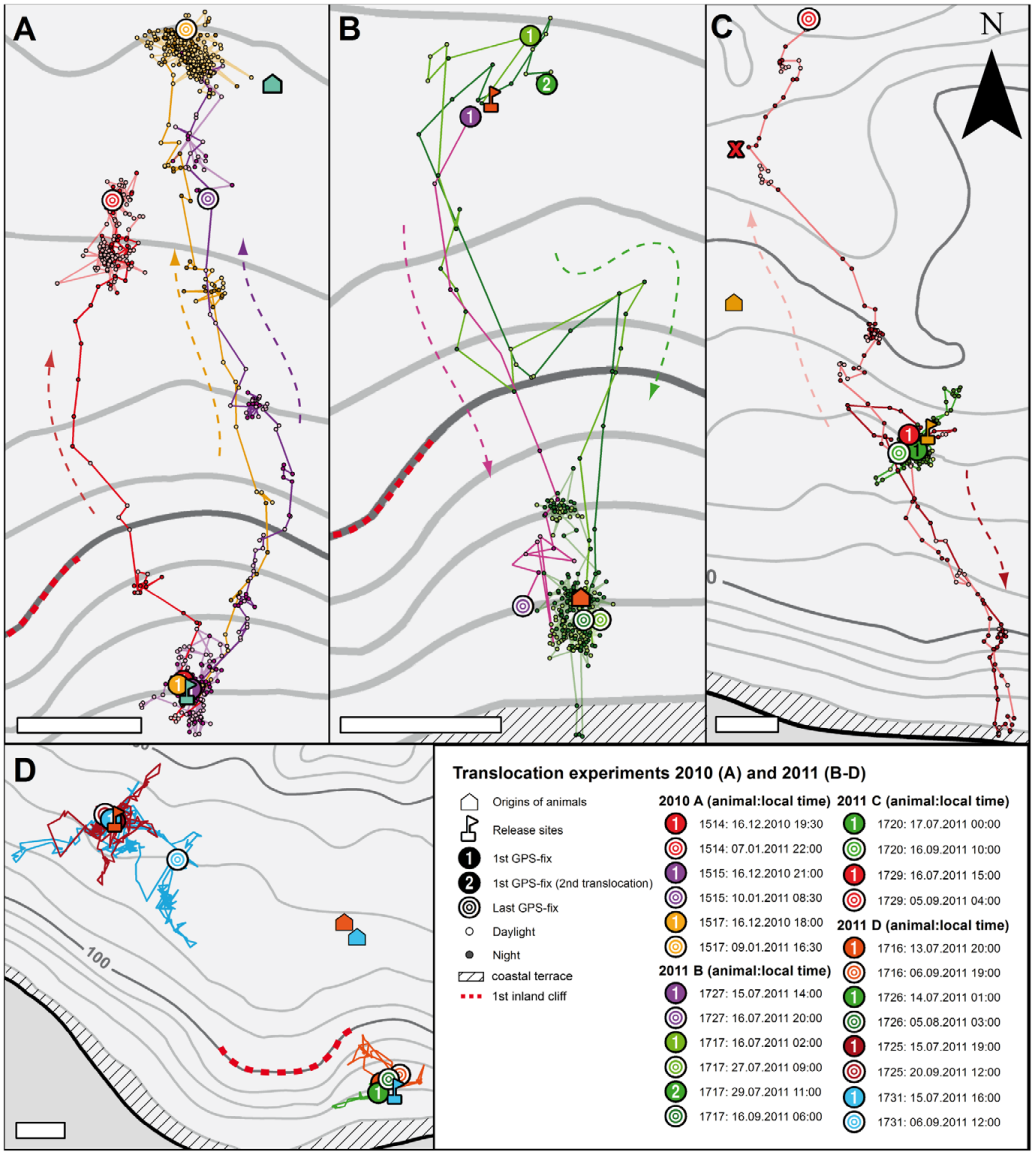
Displacement experiments and homing. **A, B:** Homing course of animals that were picked up at the house symbols and translocated in Y-axis direction towards the flagged release sites in the south (A) or the north (B). For all animals, the date (see boxed legend) and position of the first and last GPS fixes are given in addition to the track. Note that animal No. 1717 (green in B) was translocated twice and both times homed back to its pick-up site on an identical path. **C:** Two animals that were picked up at the house symbols and translocated roughly parallel to the coast out of the migratory corridor towards the flagged release sites in the east. Whereas animal No. 1720 remained in the release area, No. 1729 migrated to the coast, but ca. 1.5 km east of the regular migratory corridor. It then reversed its path to migrate far north past the release site. **D:** Two animals, Nos. 1725 and 1731, translocated roughly parallel to the coast out of the migratory corridor towards the flagged release sites in the west did not home back to the pickup area (orange symbols). Two animals translocated from north to south (blue symbols) within the migratory corridor during the 2011 mission also remained within the release area. Scale bars: 200 m.

Eight animals were translocated in a Y-axis direction (4 from north to south and 4 from south to north) with regard to the coastline (X-axis) over distances from 650 to 1,000 m within the previously identified migratory corridor (Fig. 7). The animals were carried on foot in opaque bags to prevent visual input during the translocation. Five of the translocated individuals returned to locations within a range of 100–300 m (Fig. 7A, B) of the position of capture or right to the point of capture (Fig. 7B) between 10.5 h (translocation over 650 m; No. 1727) and 21 days (translocation over 1,000 m; No. 1515) after release. Two individuals did not return to the position of capture (Fig. 7D; No. 1716, 1726) and contact with the eighth animal was lost one day after translocation. Although homing routes differed between individuals, most chose a relatively direct pathway back to their point of capture. A single individual (No. 1717) was translocated a second time after having successfully reached its capture point (Fig. 7B; light green and dark green). Interestingly, this animal returned to its capture point twice following an identical path with a characteristic S-bend. The paths taken by the other homing animals suggest that the terrain between capture and release points permitted a more direct (ca. 250 m or 27% shorter) route than that taken by No. 1717 (Fig. 7B).

**Figure 8:**
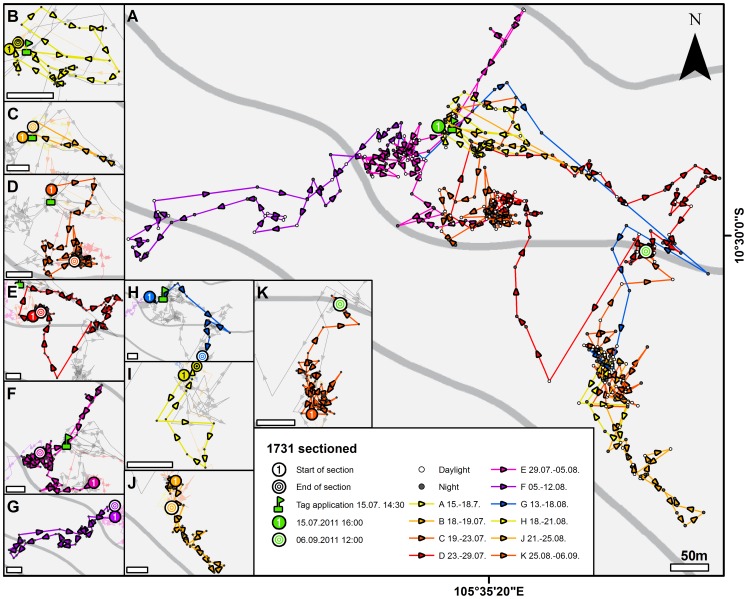
Displacement-induced search behavior. **A:** higher magnification of animal No. 1731 that was translocated roughly parallel to the coast out of the migratory corridor towards the west (compare pale blue line in Fig. 5D) and monitored from 15 July to 9 September 2011. The animal's behavior is much different from that of animals with typical site fidelity (compare Fig. 3B, C). **B–K:** Search behavior sectioned in ten migratory episodes (for dates, compare boxed legend) shows excursions in almost all compass directions. Scale bars: 50 m.

Four animals were translocated in the X-axis direction (roughly parallel to the coast) and to a similar altitude as their capture point, but to locations outside their identified migratory corridor. None of these translocated crabs successfully returned to their capture point (Fig. 7C, D). For example, animal No. 1731 did not return to its capture point in the 53 days that we were able to track it (Fig. 7D blue and 8). Notably, during this time its behavior differed significantly from that of non-translocated animals (compare Fig. 5C, C1), displaying what we interpret as search behavior. Over a period of five days, the animal undertook a series of excursions (length between 200 m and 460 m) from which it always returned to the release point (flag symbol in Fig. 8A–F, H). It then appeared to establish a second home site from which it repeated the process of exploring the nearby habitat and returning to the base point. Over a period of 53 days, the animal established 4 home sites and undertook 9 returning excursions (up to 1,200 m) before we lost contact due to battery failure. Data from the other X-axis translocation experiments support the view that for animals not capable of homing (Nos. 1725, 1731, 1720; Figs. 7C, D and 8), the release area appears to become a new reference point from which they explore the surrounding area either by wide loops that take them back to the new home or by reversing on the outbound path.

One exception was animal No. 1729 (Fig. 7C: red), which, after translocation to the east, migrated to the coast on a different path than all other animals, hitting the shoreline ca. 1.5 km east of the migratory corridor (compare Fig. 1F). It then returned on its path to migrate northwards far beyond the release site to an off-road track at the northernmost reaches of the study area. On reaching the track (X in Fig. 7C), the animal abruptly changed its route by an angle of ca. 70° and continued north along the track until we lost contact.

## Discussion

Male robber crabs display different types of movements through their habitat. They spend long periods around a home site or a resource but will perform long excursions mainly oriented toward the seashore. Translocated crabs are able to home if moved along the migratory corridor to the sea. If moved outside their migratory corridor, they are not able to relocate to their previous home site or to migrate to the stretch of coast otherwise preferred.

### Patterns of and motivation for movements

Groeneveld and Branch [7] characterized the movement of deep-water rock lobsters of the genus *Panulirus* according to three different phases: 1) nomadic – random wandering of individuals without clear start and end points, 2) homing – periodic excursions from a shelter with subsequent return or 3) migratory – seasonal long-distance movements. Using these definitions, our study indicates that *B. latro* is semi-nomadic, with phases of home site fidelity and short-distance excursions from a refuge, to phases of long-distance movements over several km. Notably, these movements occur much more frequently than previously reported by Fletcher [16]. Our GPS data show that the monitored animals are well oriented at least within their migratory corridors and that their home range typically extends from the coast to ca. 1.5 or 2 km inland. Nocturnal activity as indicated in Fig. 4 seems to be dominant in the population on Christmas Island as discussed in Drew et al. [10].

Schiller et al. [37] and a number of other authors [38]–[40] had previously reported that unlike the females, *B. latro* males do not migrate to the coast to breed. Females were thought to copulate in the inland forest and then move to coastal areas to spawn. However, tracking data from our study show that the males do move to the coast during the wet season (Fig. 6C–M), but remain there for relatively short periods of time (*N* = 8) of 4±3 days (SD, range: 1 to 10 days). This coastal movement was much more common in males tracked during the wet season than those tracked during the dry season, and data collected indicate remarkably different behavior in individuals at the coast between the two seasons. In the 2008 and 2010 monitoring (wet season), a total of 9 of the 21 males tracked were observed to migrate uni- or bidirectionally (between the coast, and the inland forest areas). This contrasts with the 14 males tracked in the 2011 dry season, of which only a single individual was observed to move to the coast from the inland forest, where it then spent a relatively protracted period of 30 days at the coast. Given the above as well as Drew's observation of a number of copulatory events on the coastal plateau in late 2009 and the discovery of a number of females with fresh spermatophores on the coastal plateau (Drew personal observations) in the 2010 breeding season, we suggest that the observed long-distance migration of males in this study may occur in a reproductive context. Schiller (1991) suggested that each female robber crab produces only one batch of eggs per year, and they presented evidence that the reproductive season at Christmas Island can last from late September to February [37]. In the wet season in 1998/1999, the peak spawning event occurred in mid-January [37]. However, in the wet seasons in 2008/2009 and 2010/2011, the spawning most likely peaked in December. If male movement to the coast is associated with reproduction we would anticipated this to occur either with, or shortly after, the primary female migration and we may have missed the largest movement of males to the coast.

Apart from reproduction, what motivates males to migrate so frequently along the Y-axis? What would be the beneficial resource at the coastal plateau that initiates long-distance migrations? Having spotted many animals hidden in blowholes on the coastal terrace during 2010, we suggest that saltwater intake in preparation for molting, which takes place at the end of the wet season (April to August; Drew personal observation), may be one of these benefits [14]. Lentic waters and especially mineral supplies are quite scarce on Christmas Island due to the porous soil and subjacent limestone [41]. Preliminary bioassays in which we offered crabs both fresh and saltwater demonstrated that contrary to previous observations [42], animals show a clear drinking preference for salt- rather than for freshwater (Hansson & Erland pers. comm.). In contrast, Greenaway (2001) confirmed that drinking saltwater is not required to maintain an ion balance as long as the animals have access to sodium-rich food sources such as red crabs [43]. Schiller [44], however, showed that females drink saltwater and that the osmolality of eggs rises drastically just before spawning. He suggests that females must drink seawater to enable egg development.

Another factor possibly affecting movements is the access to red crabs as prey. During the wet season, the annual red crab migration takes place, causing an unequal distribution of food sources. The main long-distance migratory period of *B. latro* may thus be synchronized with the annual red crab migration from the beginning of the wet season until January, when few red crabs remain on the upper and mid plateau. During the dry season in 2011, with one exception (Fig. 7C), no long-distance movements towards the coast were observed. Therefore, the increase in movement in the wet season compared to the dry season may also suggest a link with the molting period between April and August (Drew personal observations) when water and minerals are crucial [45].

If saltwater is an essential supply, why do robber crabs not permanently stay close to the coastal terrace? Conceivable explanations for the wide distribution in the inland rain forest are more favorable microclimate, avoidance of strong competition for nutrients (such as preferred plants and red crabs) and space on the coastal terrace, and presence of optimal molting sites (deep soft soil) further inland.

### Homing

Homing behavior is not only frequently observed in insects [46] but has also been demonstrated in some decapod crustaceans, both marine and terrestrial [23], [47]. The most amazing example is perhaps the spiny lobster of the genus *Panulirus,* which in Bermuda was shown to home when displaced over 2 miles [48]. Our experiments provide the first conclusive evidence for the homing abilities over large distances (up to 1 km) of a land-living crustacean, within what we assume to be the familiar territory of *B. latro*. Furthermore, our data provide detailed information on spatial and temporal aspects of homing, and demonstrate remarkable orientation abilities within their migratory corridor that provide evidence for place and route memories. The data from one animal displaced twice strongly suggest idiosyncrasy of memorized routes.

How does *Birgus* home? Chemical trail following is a well-established mechanism in insects [22], [46], but so far no indication exists for this mechanism in terrestrial crustaceans. Although emerging evidence shows that Coenobitidae, including *B. latro,* have evolved an excellent sense of aerial olfaction [21], [49], [50], our experimental data do not provide any evidence that they use this sensory modality for orientation during their long Y-axis migrations. However, route-based orientation mechanisms that rely on information stored during the outward journey might explain how the animals can navigate during excursions to the coastal terrace (route memory). In insects, route reversal provides that they memorize visual landmarks during the outbound journey [22], [46]. For the daily trans-dunal migrations over ca. 100 m from the beach to an inland bush habitat of the terrestrial hermit crab *Coenobita rugosus*, a close relative of *B. latro*, landmarks in the visual panorama were suggested to be a major cue for orientation [51]–[54]. For nocturnal arthropods such as *B. latro*, the pattern of gaps in the tree canopy, seen as visual contrasts against the sky, may also provide navigational cues [55].

We displaced animals as far as 1 km within what we consider their familiar territories, and most animals thus translocated showed robust, directed homing behavior that resulted in the animals returning close to their pick-up points. Possible information sources for orientation, all of which are known or have been discussed for malacostracan crustaceans, include celestial cues (sun, moon, anisotropic radiance distribution from skylight or reflections from the ocean or breaking surf); the earth's magnetic field; differences of substrate features; gravitational information (slope); the breeze itself (anemotaxis), which may carry ocean odors (chemotaxis); and seismic low frequency cues from breaking surf [17], [18], [23], [24], [47], [55]–[61].

Most of these features are available as orientation cues in *B. latro*'s habitat. By extracting navigational information from these cues and combining them with memorized familiar topographic features, animals may organize their migratory routes during the unforced Y-axis migrations and translocation experiments.

### Search behavior

“True navigation” describes the ability to navigate to a goal location even after displacement to unfamiliar locations outside the range of an animal's experience [62]. We interpret the search behavior that we induced in *B. latro* by X-axis translocation out of its familiar migratory corridor as if we displaced the animals into *terra incognita*. Although these animals failed to find their way back to their pick-up areas, this experiment nevertheless provided interesting insights into possible homing mechanisms to the new reference point that the release site became for these animals. During some of the outbound excursions from this central place, they simply seemed to reverse their paths for homing back. During other excursions, the animals performed wide loops as long as ca. 1 km, which nevertheless led them back to their starting points. Such observations suggest path integration is an orienting mechanism as has been suggested for fiddler crabs *Uca rapax* (Brachyura; [25], [63]–[65]). These animals make feeding excursions of up to 2 m from their burrows, during which they compute an egocentric home vector, but visual landmarks may also guide their homing behavior [25], [66]. Path integration in these animals was suggested to use, among other cues, idiothetic information, i.e. the continuous calculation of a home vector using internal measurements of locomotion such as proprioceptive signals from the legs [64], [65]. The long excursions that we observed in the X-axis translocated *B. latro* suggest that these animals rely on path integration and landmark guidance, both of which in turn require a visual/celestial or magnetic compass reference, odometry and a memory for visual scenes. It will be interesting to conduct further experiments to analyze which compass the animals rely on for their route-based orientation and if physical gradients play a role.

Our experiments reveal new aspects of robber crab life. These giant arthropods are not sessile organisms but are more or less constantly in motion, be it locally around a home site or in the context of long-distance movement. To unambiguously establish the navigational strategies used requires much more study, as has been seen in homing pigeons, where the mechanisms involved are just starting to be unraveled after decades of investigation in many laboratories. Here we reveal the first aspects of robber crab movement and navigation mechanisms using state-of-the-art technology. These data will form the basis for continued studies aimed at revealing the details of how these animals find their way around their habitat.
